# Heteronemin, a Marine Sesterterpenoid-Type Metabolite, Induces Apoptosis in Prostate LNcap Cells via Oxidative and ER Stress Combined with the Inhibition of Topoisomerase II and Hsp90

**DOI:** 10.3390/md16060204

**Published:** 2018-06-10

**Authors:** Man-Gang Lee, Yi-Chang Liu, Yi-Lun Lee, Mohamed El-Shazly, Kuei-Hung Lai, Shou-Ping Shih, Seng-Chung Ke, Ming-Chang Hong, Ying-Chi Du, Juan-Cheng Yang, Ping-Jyun Sung, Zhi-Hong Wen, Mei-Chin Lu

**Affiliations:** 1 Department of Marine Biotechnology and Resources, National Sun Yat-sen University, Kaohsiung 804, Taiwan; mg2253@yahoo.com.tw (M.-G.L.); 2 Division of Urology, Department of Surgery, Kaohsiung Armed Forces General Hospital, Kaohsiung 802, Taiwan; 3 Division of Urology, Department of Surgery, Zuoying Branch of Kaohsiung Armed Forces General Hospital, Kaohsiung 813, Taiwan; ling92@ymail.com (S.-C.K.); 4 Division of Hematology-Oncology, Department of Internal Medicine, Kaohsiung Medical University Hospital, Kaohsiung 807, Taiwan; ycliu@cc.kmu.edu.tw; 5 Department of Internal Medicine, Faculty of Medicine, College of Medicine, Kaohsiung Medical University, Kaohsiung 807, Taiwan; 6 Department of Urology, Sinying Hospital, Ministry of Health and Welfare, Tainan 730, Taiwan; leeyl05@yahoo.com.tw; 7 Department of Pharmacognosy and Natural Products Chemistry, Faculty of Pharmacy, Ain-Shams University, Organization of African Unity Street, Abassia, Cairo 115, Egypt; mohamed.elshazly@pharma.asu.edu.eg; 8 Department of Pharmaceutical Biology, Faculty of Pharmacy and Biotechnology, German University in Cairo, Cairo 114, Egypt; 9 Graduate Institute of Marine Biology, National Dong Hwa University, Pingtung 944, Taiwan; mos19880822@gmail.com (K.-H.L.); ycdu0626@gmail.com (Y.-C.D.); pjsung@nmmba.gov.tw (P.-J.S.); 10 National Museum of Marine Biology & Aquarium, Pingtung 944, Taiwan; 11 Doctoral Degree Program in Marine Biotechnology, National Sun Yat-sen University, 70 Lien-Hai Road, Kaohsiung 804, Taiwan; m6430005@hotmail.com; 12 Doctoral Degree Program in Marine Biotechnology, Academia Sinica, 128 Academia Road, Section 2, Nankang, Taipei 115, Taiwan; 13 Department and Graduate Institute of Aquaculture, National Kaohsiung Marine University, Kaohsiung 811, Taiwan; junkrough.hmc@webmail.nkmu.edu.tw; 14 Research Center for Natural Products & Drug Development, Kaohsiung Medical University, Kaohsiung 807, Taiwan; q9113054@yahoo.com.tw; 15 Chinese Medicine Research and Development Center, China Medical University Hospital, Taichung 404, Taiwan

**Keywords:** antitumor, apoptosis, autophagy, ER stress, heteronemin, Hsp90, topoisomerase II catalytic inhibitor

## Abstract

Heteronemin, a marine sesterterpenoid-type natural product, possesses diverse bioactivities, especially antitumor effect. Accumulating evidence shows that heteronemin may act as a potent anticancer agent in clinical therapy. To fully understand the antitumor mechanism of heteronemin, we further explored the precise molecular targets in prostate cancer cells. Initially, heteronemin exhibited potent cytotoxic effect against LNcap and PC3 prostate cancer cells with IC_50_ 1.4 and 2.7 μM after 24 h, respectively. In the xenograft animal model, the tumor size was significantly suppressed to about 51.9% in the heteronemin-treated group in comparison with the control group with no significant difference in the mice body weights. In addition, the results of a cell-free system assay indicated that heteronemin could act as topoisomerase II (topo II) catalytic inhibitor through the elimination of essential enzymatic activity of topoisomerase IIα expression. We found that the use of heteronemin-triggered apoptosis by 20.1–68.3%, caused disruption of mitochondrial membrane potential (MMP) by 66.9–99.1% and promoted calcium release by 1.8-, 2.0-, and 2.1-fold compared with the control group in a dose-dependent manner, as demonstrated by annexin-V/PI, rhodamine 123 and Fluo-3 staining assays, respectively. Moreover, our findings indicated that the pretreatment of LNcap cells with an inhibitor of protein tyrosine phosphatase (PTPi) diminished growth inhibition, oxidative and Endoplasmic Reticulum (ER) stress, as well as activation of Chop/Hsp70 induced by heteronemin, suggesting PTP activation plays a crucial rule in the cytotoxic activity of heteronemin. Using molecular docking analysis, heteronemin exhibited more binding affinity to the N-terminal ATP-binding pocket of Hsp90 protein than 17-AAG, a standard Hsp90 inhibitor. Finally, heteronemin promoted autophagy and apoptosis through the inhibition of Hsp 90 and topo II as well as PTP activation in prostate cancer cells. Taken together, these multiple targets present heteronemin as an interesting candidate for its future development as an antiprostatic agent.

## 1. Introduction

Cancer is one of the leading causes of death worldwide. The global estimates of cancer prevalence for 27 sites in the adult population indicated the five-year global cancer prevalence to be 28.8 million in 2008. Different regions are denominated with certain types of cancers, but breast cancer remains the most prevalent cancer globally. Prostate cancer (PCa) is a significant cause of death in northern and western Europe, North America, and Oceania. It is considered the sixth leading cause of male cancer death in Western developed countries [1]. PCa prognosis can be serious due to the high probability of bone metastases that occur in over 80% patients with advanced PCa. In the last few years, more than 200,000 new cases of PCa were diagnosed with more than 30,000 deaths due to drug resistance to the US’s Food and Drug Administration (FDA)-approved first-line of treatment, the semisynthetic natural product docetaxel [2,3]. With the aging population, the development of more effective and safer drugs to treat advanced metastatic castration-resistant prostate cancer is an urgent need. Nature and its untapped treasures of natural products from terrestrial and marine organisms remains the source of inspiration for drug leads for prostate cancer and other types of cancer.

Oceans cover over 70% of the earth surface and its complex ecosystems offer vast biodiversity [4]. Marine organisms are a treasure trove of secondary metabolites with their unique skeletons and diverse biological activities including anticancer, anti-inflammatory, antimicrobial, and antioxidant activities [5,6,7]. Among these activities, the anticancer effect of marine secondary metabolites is considered the most interesting activity due to the unprecedented potency of marine compounds against several types of cancers [8]. Certain groups of marine secondary metabolites proved to exert potent anticancer activities including alkaloids, terpenes, peptides, and steroids [9].

Scalarine sesterpenoids attracted attention due to their diverse biological properties, which encouraged scientists to modify their skeleton to produce more potent derivatives [10]. In 2012, our group isolated pentacyclic sesterterpenes and scalarane-type sesterterpenes from sponge *Hippospongia* sp. [11]*.* One interesting example of this class of secondary metabolites is heteronemin, which was isolated from the marine sponge *Hyrtios* sp. in 1994 and showed potent cytotoxic activity [12]. It was also investigated for other biological activities including antifeedant, antimicrobial ichthyotoxic, protein inhibitory, and antitubercular activities [13]. Heteronemin possesses a pentacyclic scalarane skeleton including dihydrofuran moiety [10]. For the privileged cytotoxicity, this marine natural product demonstrated significant inhibition of viability and proliferation against a series of cancer cell lines, including leukemia, colon and breast cancer, cervica,l and renal carcinoma with IC_50_ less than 1 micromolar [11,14,15].

Schumacher et al. indicated that this sponge sesterterpene inhibited TNFα-induced NF-κB activation through proteasome and induced apoptotic cell death [16]. Such findings are interesting because compounds targeting hypoxic signaling in tumors or phospholipases A2 in inflammatory diseases represent potential therapeutic agents [4,17]. Heteronemin exhibited potent cytotoxic activity against prostate cancer LNcap cells with IC_50_ values of 1.4 μM. However, the precise cytotoxic action of this natural product has not been elucidated. In the study, we investigated the cytotoxic and antitumor effects of heteronemin as well as its mechanism of action in human prostate cancer cell lines with androgen-dependent and independent types in vitro cellular and in vivo xenograft models.

## 2. Results

### 2.1. Effect of Heteronemin on Cellular Viability In Vitro Assay

**Figure 1:**
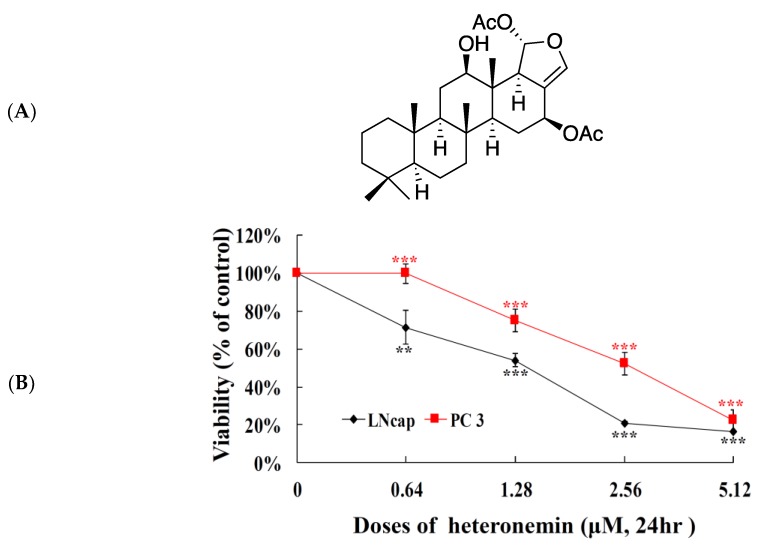

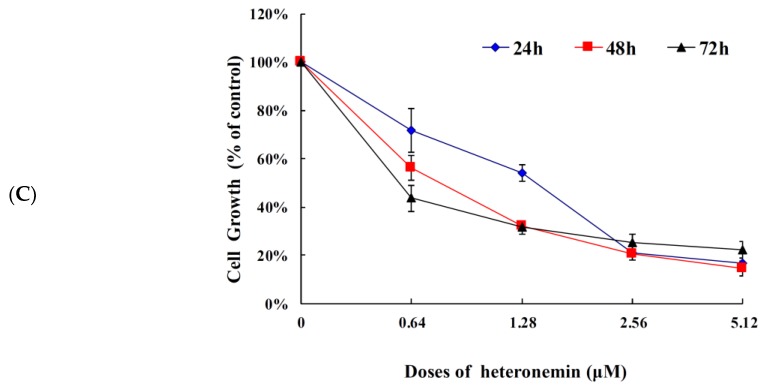
Effect of heteronemin (**A**) on (**B**) cellular viability of human prostate cancer LNcap and PC3 cells in a dose-dependent manner for 24 h and (**C**) cellular growth of human prostate cancer LNcap cells in a time-dependent manner for 24, 48 and 72 h, respectively. Results are shown as mean ± SD (Standard Deviation) of three independent experiments. Data are expressed as mean ± SD. ** *p* < 0.01 or *** *p* < 0.001 vs solvent group.

In our previous report, the antiproliferation of heteronemin (Figure 1A) was significant on a panel of human cancer cell lines including DLD-1 (human colon adenocarcinoma), HCT-116 (human colorectal carcinoma), K562 (human chronic myelogenous leukemia), and T-47D (human breast ductal carcinoma) cancer cells. Incubation of different cancer cells with heteronemin for 72 h resulted in the suppression of cancer cells viability with IC_50_ <0.002 μM [11]. The potent cytotoxic activity of heteronemin against several cancer cell lines encouraged us to evaluate its activity against prostate cancer cells which is the 2nd most commonly diagnosed cancer [1]. The cytotoxic activity of heteronemin was evaluated using MTT [3-(4,5-cimethylthiazol-2-yl)-2,5-diphenyl tetrazolium bromide] assay against human prostate cancer cell lines, including LNcap and PC 3 cells, for 24 h. As shown in Figure 1B, heteronemin inhibited the growth of LNcap and PC 3 cells in a dose-dependent manner. The IC_50_ values of heteronemin were 1.4 and 2.7 μM, respectively suggesting that LNcap cells are more sensitive toward heteronemin compared with PC3 cells. The effect of heteronemin on cell viability of LNcap cells over different time intervals was also examined. The growth inhibition of heteronemin was determined after 24, 48 and 72 h in LNcap cells resulting in IC_50_ values of 1.4, 0.8 and 0.4 μM, respectively (Figure 1C).

### 2.2. Effect of Heteronemin on the Growth of Human Prostate LNcap Tumor In Vivo Xenograft Animal Model

**Figure 2:**
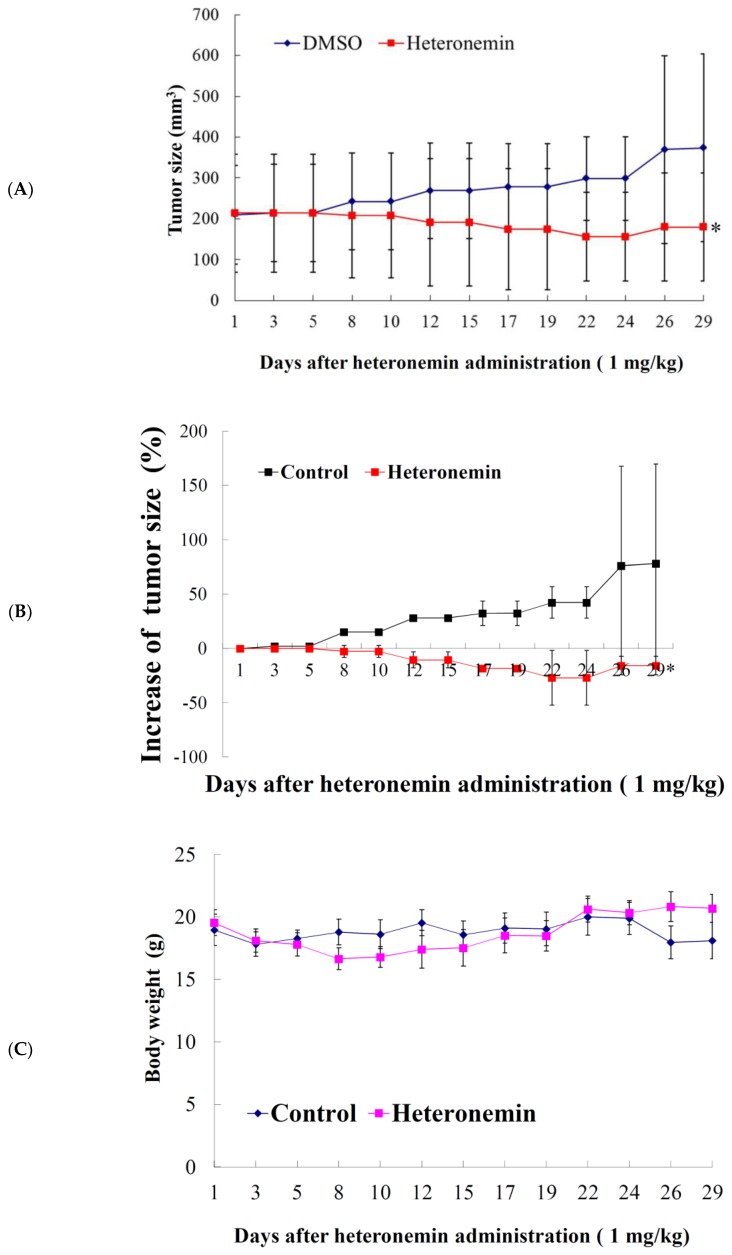
Effect of heteronemin on tumor growth and body weight in vivo LNcap tumor xenograft animal model. Tumor-bearing nude mice were intraperitoneally injected with solvent control (DMSO) and heteronemin (1 mg/kg of body weight) for 29 days. (**A**) Tumor volumes were measured every other day, and the results are expressed as mean ± SD. * Significantly different from the control groups at * *p* = 0.029. (**B**) Effect of heteronemin on the increase of tumor size. The curve of tumor growth was normalized to the starting volume. * Significantly different from the control groups at * *p* = 0.018. (**C**) The body weight was measured every other day, and the results are expressed as mean ± SD. Control, *n* = 8; heteronemin, *n* = 7.

Our in vitro results showed that heteronemin treatment could suppress the proliferation of LNcap and PC3 cells. However, LNcap cells line was more sensitive to heteronemin treatment. The in vivo antitumor activity of the compound was further determined by evaluating its effect on the tumor growth of human prostate LNcap in xenograft animal model. LNcap (1 × 10^5^) cells were inoculated subcutaneously at the right flank of male immunodeficient athymic mice. After 29 days of treatment, the tumor growth was significantly suppressed under the influence of intraperitoneal injection of heteronemin (1 mg/kg of body weight). The average tumor size on day 29 in the control group was 373.8 mm^3^, whereas the average tumor size in the heteronemin-treated group was 179.9 mm^3^. The tumor size was significantly reduced in the treated group by 51.9% as compared with the control group (*p* = 0.029) with no significant difference in the mice body weights (Figure 2A,C). The tumor size gradually increased in the control group to 76.1% in comparison with the starting volume. On the other hand, the tumor size decreased in the heteronemin-treated group to −15.8% over the course of this study, in comparison with the starting volume (Figure 2B) (*p* = 0.018). The decrease in the tumor volume and the inhibition of tumor growth demonstrated that heteronemin exhibited the antitumorigenic effect in vivo xenograft model.

### 2.3. Effect of Heteronemin on Apoptotic and Autophagic Induction in LNcap Cells

**Figure 3:**
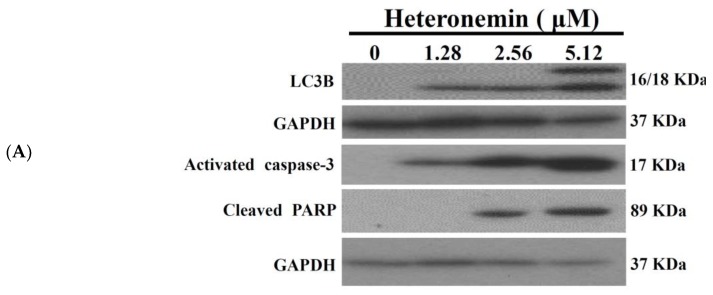

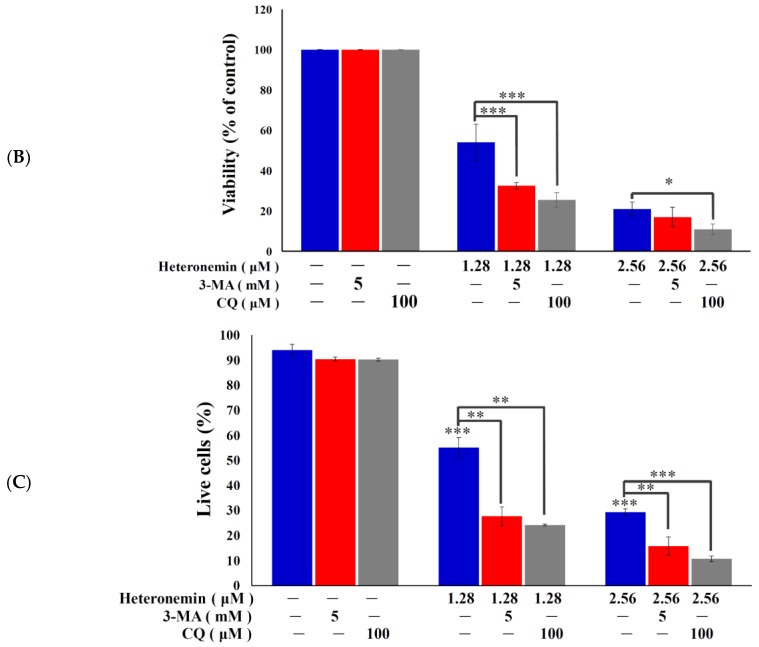
Effect of heteronemin on the induction of apoptosis and autophagy in LNcap cells. (**A**) LNcap cells were treated with heteronemin (0, 1.28, 2.56 and 5.12 μM) for 24 h. The protein expression of activated caspase 3 and cleaved PARP, as well as LC3B II, was analyzed by Western blotting. The GAPDH (Glyceraldehyde-3-Phosphate Dehydrogenase) was the loading control. The viability (**B**) and apoptosis (**C**) of LNcap cells were determined with MTT and flowcytometric analysis after treatment of heteronemin (blue bars), heteronemin + 3-MA (red bars) and heteronemin + CQ (grey bars). Data are expressed as mean ± SD. * *p* < 0.05; ** *p* < 0.01; and *** *p* < 0.001 vs heteronemin group.

Recent evidence indicated that compounds which cause apoptotic induction may act as potential anticancer drug candidates [18]. The marine sesterterpenoid, heteronemin, was found to induce apoptosis and autophagy in human renal carcinoma cells [14]. In the current study, we attempted to identify the precise mechanism by which heteronemin mediated the death of LNcap cells. Hallmarks of apoptosis [cleavage of PARP (Poly ADP-Ribose Polymerase) and caspase 3] and autophagy (activation of LC 3B II) were determined by Western blotting analysis. We treated LNcap cells with different concentrations of heteronemin for 24 h. In agreement with the previous study [19], heteronemin treatment caused a dose-dependent upregulation of caspase 3 and cleavage of PARP as well as activation of LC3-II, which confirms the induction of apoptosis and autophagy (Figure 3A). The functional relationship between autophagy and apoptosis is complex and there are multiple connections and cross regulations between the two processes. Certain stimuli can trigger both processes. However, both autophagy and apoptosis can be developed in mutually exclusive manner in response to variable thresholds for the two processes or because of the cellular selection between the two responses that may end up in mutual inhibition of both processes [20]. In general, autophagy precedes apoptosis and autophagy impedes the induction of apoptosis. On the other hand, apoptosis-associated caspase activation switches off the autophagic process [21]. Consequently, our result showed that the induction of the autophagy (expression of LC-3 II) reached its peak after treatment with 1.28 and 2.56 μM of heteronemin, but autophagy was suppressed by apoptosis after the treatment with 5.12 μM of heteronemin resulting in the induction of LC-3 I expression and caspase activation. To investigate whether autophagy was involved in the cytotoxicity of heteronemin, LNcap cells were pretreated with the early autophagy inhibitor, 3-MA, or the late autophagy inhibitor, chloroquine (CQ). Significant differences were observed in the groups pretreated with 3-MA or CQ in comparison with the heteronemin group. The viability and living cells population of heteronemin + 3-MA or heteronemin + CQ were reduced compared with the heteronemin group, suggesting that 3-MA and CQ enhanced the cytotoxicity and autophagy induction of heteronemin (Figure 3B,C). In addition, these result demonstrated that autophagy possessed the cytoprotective effect, and attenuation of autophagy decreased the viability and increased the apoptosis induction in heteronemin-treated LNcap cells. The above results suggested that the cytotoxic effect of heteronemin on human prostate LNcap cells was associated apoptotic cell death.

### 2.4. Effect of Heteronemin on Topoisomerase I and II Activity

**Figure 4:**
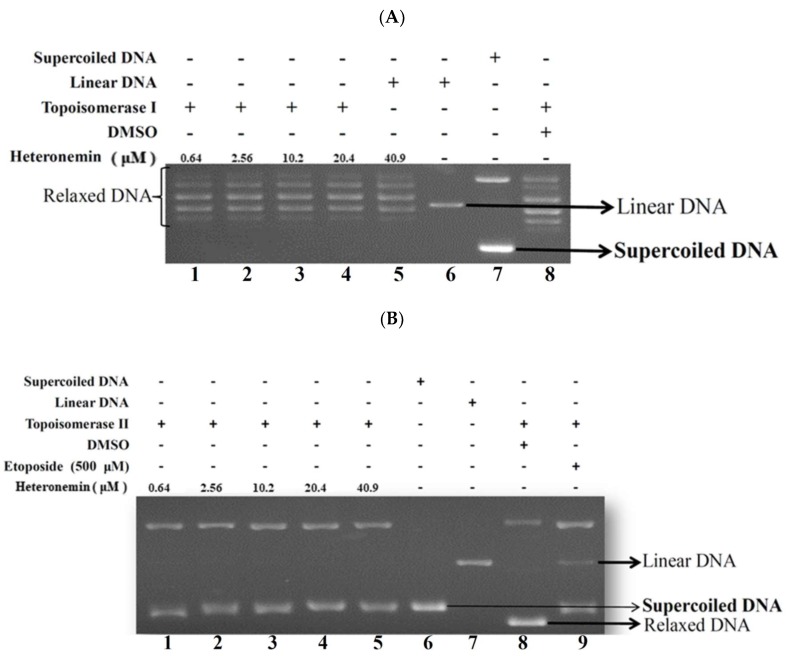

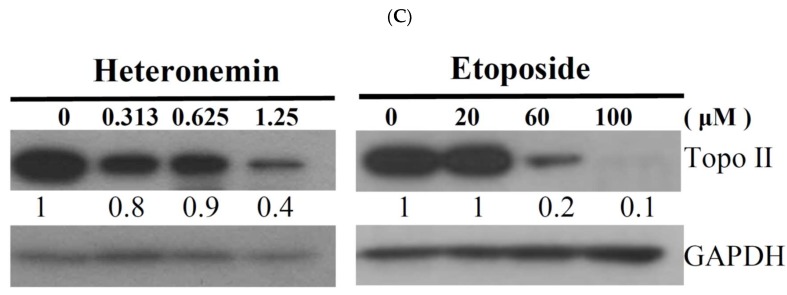
Effect of heteronemin on topo I and II activity. (**A**) Effect of heteronemin on topo I activity. Lanes 1–5: heteronemin (0.64, 2.56, 10.2, 20.4, and 40.9 μM); Lane 6: Linear DNA; Lane 7: negative control plasmid DNA (supercoiled DNA); Lane 8: plasmid DNA + topo I + solvent control (induction of DNA relaxation); (**B**) effect of heteronemin on topo II activity. Lanes 1–5: heteronemin (0.64, 2.56, 10.2, 20.4, and 40.9 μM); Lane 6: negative control plasmid DNA (supercoiled DNA); Lane 7: Linear DNA; Lane 8: plasmid DNA + topo II + solvent control (induction of DNA relaxation). Lane 9: positive control, etoposide (500 μM), as topo II poison (induction of linear DNA). (**C**) Heteronemin and etoposide treatment decreased the expression of topo IIα protein in LNcap cells. Cells were treated with heteronemin (0, 0.64, 1.28 and 2.56 μM) and etoposide (0, 20, 60 and 100 μM) for 24 h, respectively. Protein expression was analyzed by Western blotting. The bands were quantified via densitometry and normalized relative to the GAPDH levels.

Recent investigation suggested that inhibitors of DNA topoisomerase are of great therapeutic interest because they help to maintain strand breaks generated by topoisomerases during replication [22]. Topo II is involved in DNA replication, transcription and chromosome segregation, which are upregulated in highly proliferative cancer cells [23]. Camptothecin and its derivatives which interfere with topo I activity are used to treat colorectal, ovarian and lung cancers, while few other topo I inhibitors are being tested in clinical trials [22]. Topo II poisons induce DNA cleavage. Several important classes of anticancer drugs may act as topo II poisons, including the non-DNA intercalator, etoposide and the DNA intercalator, doxorubicin [24]. Certain secondary metabolites were found to inhibit topoisomerase activity including the marine sesterterpenoid analogs, manoalide-25-acetals, which inhibited the DNA-relaxing activity of mouse DNA topo I and the DNA unknotting activity of calf thymus topo II [12]. To evaluate heteronemin effect on topo I and II activity and to understand its actual cytotoxic mechanism of action, a cell-free DNA cleavage assay using an enzyme-mediated negatively-supercoiled pHOT1 plasmid DNA was applied. As shown in Figure 4A, the result of the cell-free system revealed that the activity of topo I was not affected by heteronemin treatment, even at the highest concentration (40.9 μM). However, the use of heteronemin (more than the concentration at 2.56 μM) inhibited DNA relaxation by 98% compared with the control and resulted in the formation of supercoiled DNA products in the presence of topo IIα (Lanes 2–5) (Figure 4B). A linear DNA strand was observed upon treating the supercoiled pHOT1 plasmid DNA with etoposide, a standard topo II poison (Lane 9) [25]. Heteronemin treatment interrupted the process of topo II catalytic cycle and could be used as a potent catalytic inhibitor of topo II.

A reduction in the related proteins’ expression is reflected in the suppression of the overall catalytic activity. It is proposed that compounds with catalytic inhibitory activity kill cells by elimination the essential enzymatic activity of topo II [25]. Western blotting analysis was performed to determine the change of topo II proteins expression. Western blotting analysis indicated that the use of heteronemin at different doses (0.64, 1.28 and 2.56 μM) and etoposide at different doses (20, 60 and 100 μM) significantly diminished topo IIα proteins expression by 19, 11, and 61% as well as 1, 23 and 96%, respectively, in comparison with the control group. In addition, Western blotting indicated that the use of heteronemin (1.25 μM) significantly diminished topo IIα protein expression, nevertheless, etoposide predominantly reduced the expression at 60 μM. Furthermore, heteronemin and etoposide inhibited topo II activities with IC_50_ of 1.1 and 50.0 μM, respectively as demonstrated by the Western blotting assay (Figure 4C). These results suggested that heteronemin could act as a potent inhibitor of topo II.

### 2.5. Apoptotic Induction of Heteronemin Involves Disruption of Mitochondrial Membrane Potential (MMP) in LNcap Cells

**Figure 5:**
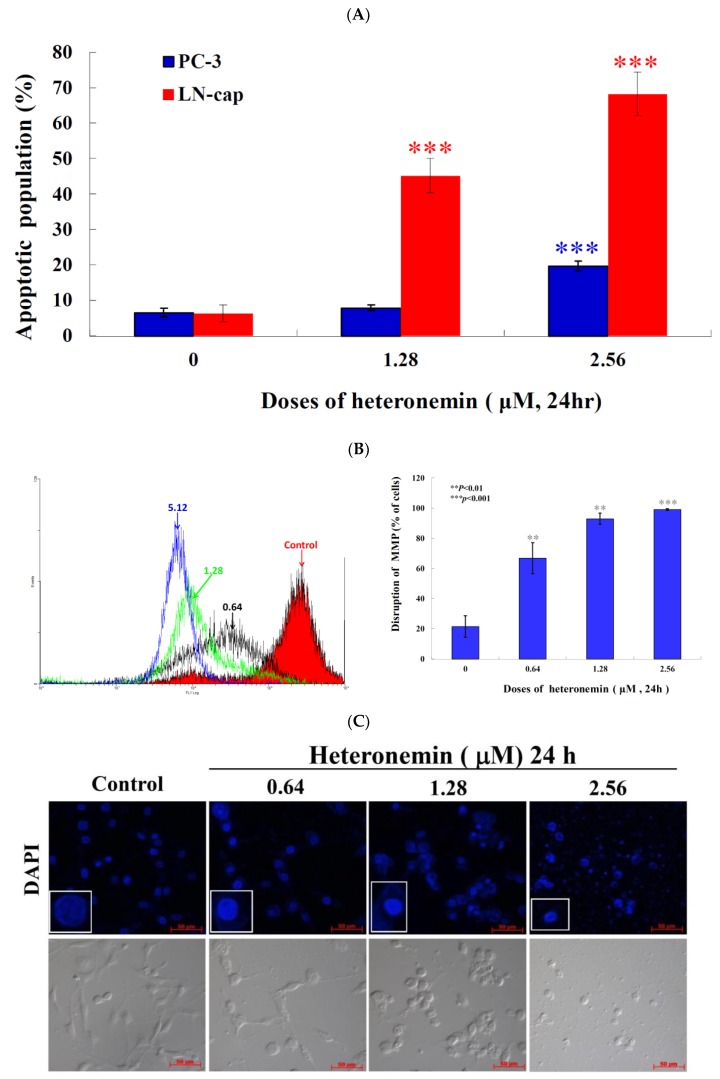
Heteronemin induces apoptosis and MMP disruption in LNcap cells. Cells were treated with the indicated concentrations of heteronemin for 24 h. (**A**) Apoptosis induction of LNcap and PC3 cells as well as (**B**) mitochondrial membrane potential of LNcap were assessed with annexin V/PI and rhodamine 123 staining using flow cytometric analysis; (**C**) the change of nuclear morphology was determined with DAPI staining and observed in LNcap cells using a fluorescent microscope. The results are presented as mean ± SD of three independent experiments (** *p* < 0.01; *** *p* < 0.001).

Previous studies demonstrated a significant cytotoxic effect of heteronemin against several cancer cell lines [10,11,26]. In the current investigation, we found that heteronemin induced LNcap cells apoptosis (Figure 3). To better comprehend the antitumor mechanism of heteronemin, the apoptotic cells population was determined using annexin V/PI and DAPI (4′,6-Diamidino-2-phenylindole) staining of LNcap cells and the effect was observed by flow cytometry and microscope. As indicated in Figure 5A, after 24 h of treatment (1.28 and 2.56 μM), the percentage of apoptotic cells population was significantly increased by 45.1% and 68.3% as well as 7.9% and 19.7% in LNcap and PC3 cells compared with the solvent control, respectively. To address whether the apoptotic induction of heteronemin was related to mitochondrial pathway, rhodamine 123 fluorescent dye was used to determine MMP. LNcap cells were treated with different doses of heteronemin for 24 h and then stained with rhodamine 123. As shown in Figure 5B, the use of heteronemin (0.64 μM) increased the population of LNcap cells with disrupted MMP to 66.9%. This effect was significantly increased upon the treatment with heteronemin at 1.28 and 2.56 μM, which resulted in 93.0% and 99.1% cells with disturbed MMP, respectively. To assess the nuclear morphological change induced by heteronemin, LNcap cells were further examined with DAPI staining and observed under a fluorescence microscope. The results showed that heteronemin treatment increased the number of condensed nuclei compared with the control, which showed intact and normal nuclei (Figure 5C). To further explore the antitumor mechanism of heteronemin, the effect of heteronemin on mitochondrial metabolism-related proteins was evaluated.

### 2.6. Effect of Heteronemin on Reactive Oxygen Species (ROS) Generation and ER Stress in LNcap Cells

**Figure 6:**
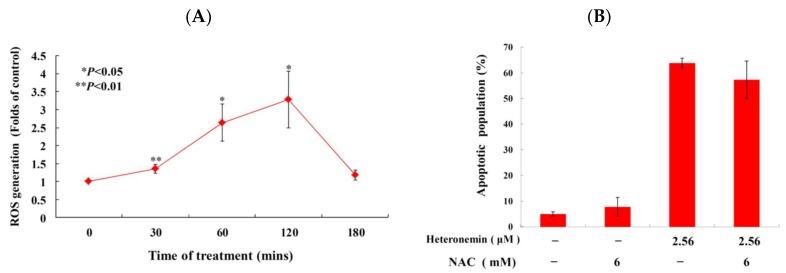

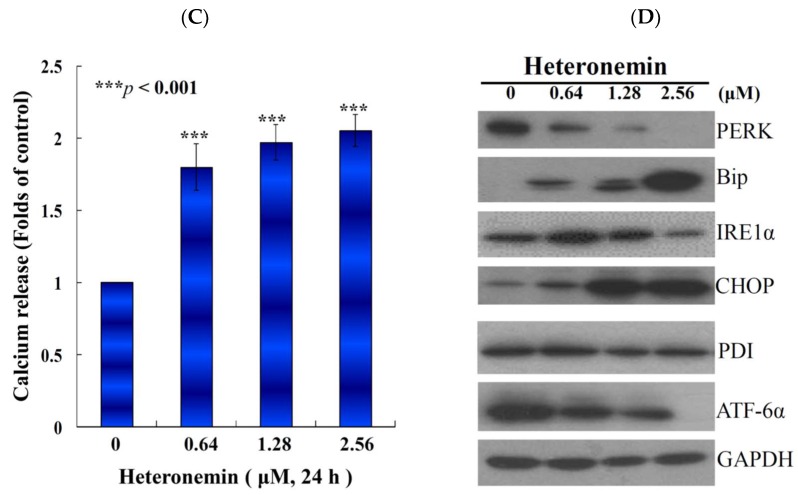
Effect of heteronemin on ROS generation and ER stress in LNcap cells. (**A**) Cells were treated with heteronemin (2.56 μM) for the indicated times. Quantitative results showed a gradual increase in the ROS production in response to heteronemin treatment when compared with the control group. (**B**) The effect of ROS generation on heteronemin-induced apoptosis in LNcap cells was examined. Cells were pretreated with 6 mM of NAC for 2 h, then treated with 2.56 μM of heteronemin. The apoptotic population was examined with annexin-V/PI assay. The results are presented as mean ± SD of three independent experiments (* *p* < 0.05; ** *p* < 0.01). (**C**) Effect of heteronemin on ER stress in LNcap cells. Cells were treated with different doses of heteronemin (0, 0.64, 1.28 and 2.56 μM) for 24 h. Quantitative results showed a gradual increase in the folds of calcium release in response to heteronemin treatment when compared with the control group. The results are presented as mean ± SD of three independent experiments (*** *p* < 0.001). (**D**) The effect of heteronemin on the expression of ER stress-related proteins of LNcap cells was examined with Western blotting assay. Cells were treated with the different doses of heteronemin (0, 0.64, 1.28 and 2.56 μM) for 24 h.

We also examined whether the antitumor effect of heteronemin involves the overproduction of ROS. The levels of ROS at different times following treatment with heteronemin were determined. A time-dependent increase in ROS generation was monitored using a carboxy derivative of fluorescein, carboxy-H_2_DCFDA dye. As shown in Figure 6A, heteronemin treatment (2.56 μM) for 30, 60, 120 and 180 min resulted in 1.4-, 2.6-, 3.3- and 1.2-fold increase in the ROS levels, respectively, as compared with the mean fluorescence index (MFI) of the control. To clarify whether ROS generation is the major regulatory factor in the apoptotic induction of heteronemin, LNcap cells were pretreated with 6 mM of *N*-acetyl-l-cysteine (NAC), an ROS scavenging agent, aiming to suppress the intracellular oxidative stress [27]. The apoptotic population was measured via annexin V/PI staining after heteronemin treatment. As shown in Figure 6B, the result of NAC pretreatment is similar to the negative control group, showing less than 10% of the apoptotic population. In addition, the pretreatment with 6 mM of NAC only attenuated 6.5% of the apoptotic cell population in response to the use of 2.56 μM of heteronemin. These results indicated that blocking the oxidative stress by NAC did not result in saving LNcap cells from apoptosis induced by heteronemin.

As previously demonstrated, calcium homeostasis and endoplasmic reticulum (ER) stress are major components in the control of autophagy and apoptosis in cancer therapy [28]. The opening of permeability transition pore in the mitochondrial inner membrane was reported to trigger the discharge of calcium into the mitochondria [29]. Fluo-3 AM is a fluorescence indicator of intracellular calcium and it used to measure Ca^2+^ inside living cells with flow cytometric analysis. To study if the antitumor effect of heteronemin involves disruption of calcium concentration, we examined calcium concentration with Fluo-3 staining after treatment with heteronemin. As shown in Figure 6C, Ca^2+^ concentration increased to 1.8-, 2.0-, and 2.1-folds compared with the solvent control after heteronemin treatment at 0.64, 1.28 and 2.56 μM, respectively, for 24 h.

Disrupted ER stress-related processes such as the unfolded protein response (UPR) and calcium homeostasis were reported as targets for anticancer agents [30]. Our results suggested that heteronemin treatment disrupted calcium homeostasis (Figure 6C). It is known that the depletion of calcium in ER also disturbs the function of ER chaperones and triggers ER stress, leading to the activation of the unfolded protein responses [30,31]. To investigate if heteronemin effect on LNcap cells involves these targets, three major transmembrane transducer proteins, PERK (Protein kinase R-like Endoplasmic Reticulum Kinase), ATF 6 (Activating Transcription Factor 6) and IRE 1α (Inositol-Requiring Enzyme-1α), as well as ER chaperone, GRP78 (78 kDa Glucose-Regulated Protein) and CHOP (CCAAT-enhancer-binding protein Homologous Protein) were determined with Western blotting assay. Our results showed that heteronemin treatment suppressed the expression of ATF 6 and PERK, while the expression of IRE 1α, Bip and CHOP was significantly increased in a dose-dependent manner after the treatment with the indicated doses of heteronemin (Figure 6D).

### 2.7. Antitumor Effect of Heteronemin Involves Protein Tyrosine Phosphatases PTP Activation 

**Figure 7:**
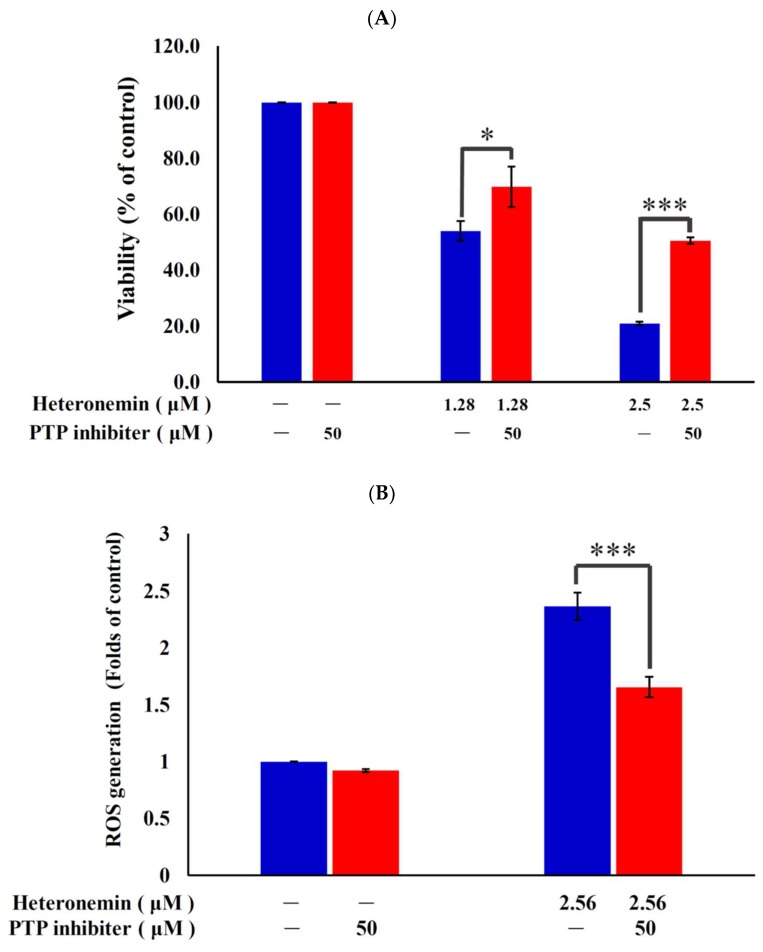

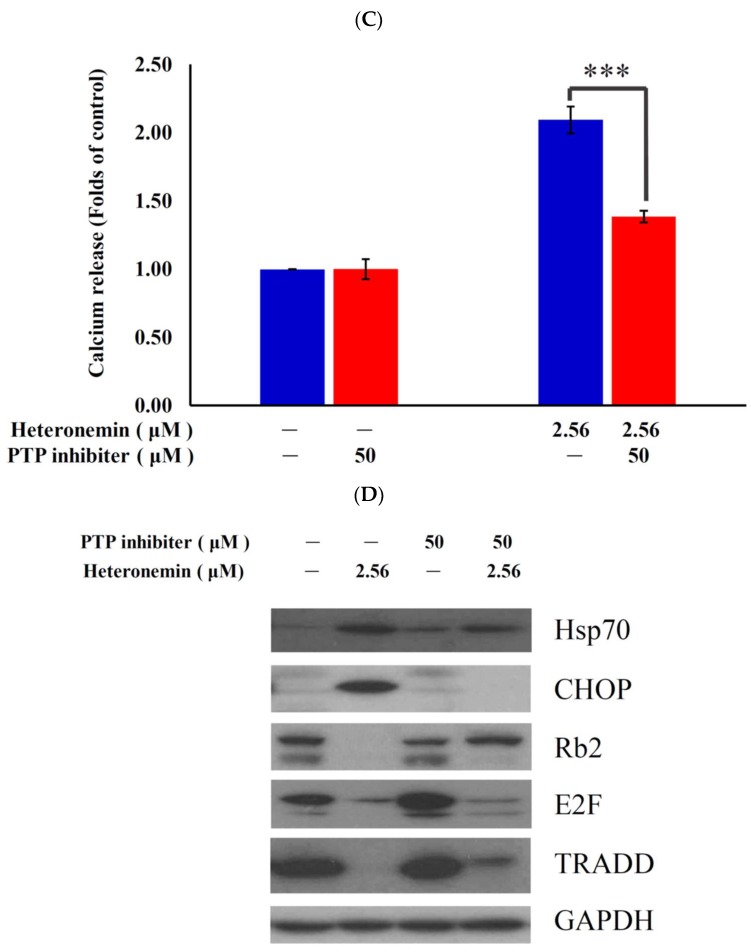
The antitumor effect of heteronemin in LNcap cells involves PTP activation. We evaluated the effect of the heteronemin treatment on growth inhibition and ROS generation in LNcap cells. Cells were pretreated with 50 μM of PTP inhibitor for 2 h (red bars) and then treated with 2.56 μM of heteronemin. Cell growth (**A**), ROS generation (**B**), calcium accumulation (**C**) and protein expression (**D**) were examined with MTT assay, carboxy-H_2_DCFDA, Fluo-3 dye staining and western blotting assays. Results are presented as mean ± SD of three independent experiments (* *p* < 0.01; *** *p* < 0.001).

Recent studies indicated that protein tyrosine phosphatases (PTPs) are a large and multifunctional family of proteins, which regulates cell proliferation, differentiation, survival, and apoptosis [32,33]. To verify whether the antitumor effect of heteronemin involves PTP activation, the PhenylHydrazonoPyrazolone Sulfonate 1, which acts as a reversible, active-site targeting, substrate-competitive inhibitor of Shp-2 [34], was used to evaluate the antiproliferation, ROS generation and ER stress in cells-treated with heteronemin. The pretreatment with 50 μM of PTP inhibitor increased the viability from 54.0% and 20.9% to 69.8% and 50.6% in response to the use of 1.28 and 2.56 μM of heteronemin, respectively (Figure 7A). These results indicated that blocking the activation of PTP by a specific inhibitor resulted in saving LNcap cells from death induced by heteronemin. To further confirm if ROS overproduction induced by heteronemin is initiated by PTP activation, the population of the cells with disturbed ROS generation in response to heteronemin treatment with or without pretreatment of PTP inhibitor was determined. The determination of the increase of mean fluorescence index (MFI) with ROS generation was achieved utilizing a specific dye, dichloro-dihydro-fluorescein diacetate (DCFHDA). The pretreatment with 50 μM of the specific inhibitor, PhenylHydrazonoPyrazolone Sulfonate 1, significantly attenuated ROS generation and calcium accumulation from 2.4 to 1.7 folds and 2.1 to 1.4 folds in response to the use of 2.56 μM of heteronemin, respectively, as compared with MFI of the control. These findings suggested that blocking the activation of PTP suppressed ROS generation and ER stress induced by heteronemin (Figure 7B,C). Moreover, PTPi pretreatment reversed the elevation of CHOP and Hsp70 as well as suppression of Rb2, E2F and TRADD induced with heteronemin treatment (Figure 7D). In agreement with the preceding results of MTT assay, these findings indicated that the antitumor effect of heteronemin in LNcap cells is mediated through apoptosis induction as well as oxidative and ER stress involving PTP activation.

### 2.8. Heteronemin Acts as Potent Hsp90 Inhibitor

**Figure 8:**
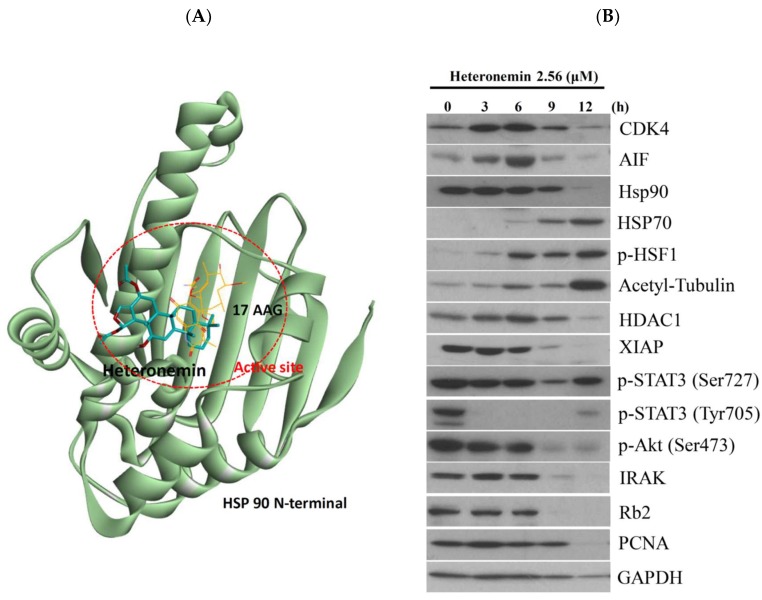

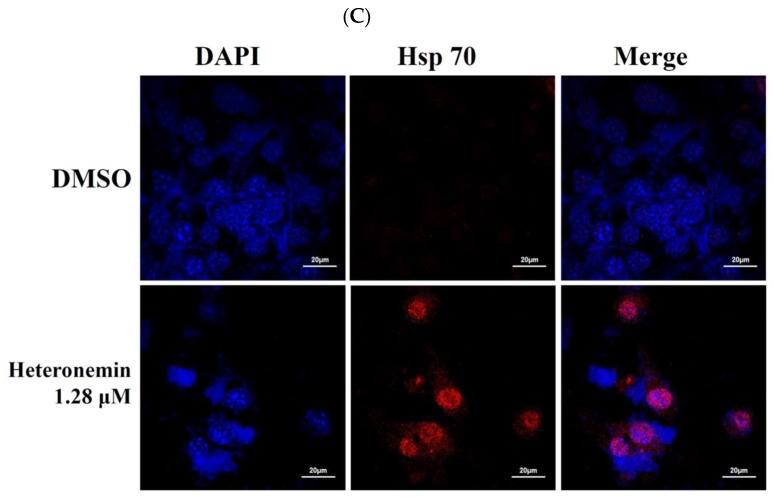
Heteronemin as the potent inhibitor of Hsp90. (**A**) Molecular modeling of Hsp90 protein with heteronemin as assessed by Autodock 4.2 software with Lamarckian Genetic Algorithm. (**B**) Effect of heteronemin on the expression of Hsp90 client proteins. (**C**) Effect of heteronemin on localization of Hsp70 protein.

A previous study indicated that heteronemin suppressed STAT3 (Signal Transducer and Activator of Transcription 3), which in turn inhibited the phosphorylation of Src/STAT3 signaling pathway. This effect led to the disruption of the nuclear accumulation and transcriptional activity of STAT3 [19]. Another report also indicated that the antitumor effect of luteolin, a plant flavonoid, involved the suppression of the phosphorylation (Tyr705) of STAT3 through binding of Hsp90 to STAT3 to promote its interaction to SHP-1 in gastric cancer cells [35]. We observed that the activation of protein tyrosine phosphatases is involved in the antitumor effect of heteronemin in LNcap cells (Figure 7). To better elucidate the antitumor potency of heteronemin at the molecular level and to understand the structural basis of its binding mode, a docking study of these compounds was performed using Autodock 4.2 with Lamarckian Genetic Algorithm. The results of the docking studies using AutoDock 4.2 showed that heteronemin binds to the active site region of Hsp90 with good binding energy −8.97 kcal/mol. In our model, heteronemin interacts with ARG 46, GLU 47, and PHE 138 (Figure 8A). The hydroxy group forms a hydrogen bond with GLU 47 and the carbonyl oxygen atom on the acetyl group attached to the five-membered ring forms another hydrogen bond with ARG 46. Despite that, the 3,3-dimethylcyclohexyl ring of heteronemin was located in the hydrophobic cavity of Hsp90 and formed proximal contacts with Phe138, the π–π interactions between 3,3-dimethylcyclohexyl ring and Phe138 was modest. The angle and the distance between 3,3-dimethylcyclohexyl ring and the rest of the compound did not seem to be optimally shaped to negate the proximal π–π contact between 3,3-dimethylcyclohexyl ring and Phe138. All these interactions contributed to a calculated inhibition constant (K_i_) 266.9 nM (>15.28 folds) which was more potent than 17-AAG (4.03 μM).

To further identify if the antitumor effect of heteronemin involves inhibition of Hsp90, we examined the expression of its client proteins using Western blotting assay. Aiming to understand the relation between Hsp90 function and the expression of Hsp90 clients, we treated LNcap cells with heteronemin (2.56 μM) at different time intervals. In agreement with our previous studies [36], heteronemin treatment suppressed the expression of Hsp90 protein along with the stimulation of Hsp70 expression, tubulin acetylation, and Heat Shock Factor 1 (HSF1) phosphorylation in a time-dependent manner. As expected, the suppression of Hsp90 client proteins was observed, including IRAK1, *p*-Akt (Ser473) and XIAP (prosurvival proteins), Rb2 (oncoproteins), HDAC1, PCNA and CDK4 (cell cycle regulatory protein), phosphorylation of STAT3 (Ser727 and Tyr705) (transcription factor) (Figure 8B). Furthermore, we identified the localization of Hsp70 in response to heteronemin treatment using immunofluorescence assay by confocal microscope. In agreement with the Western blotting results, the localization of Hsp70 was predominantly accumulated in the cytosol and nuclear compartments (Figure 8C). Taken together, our molecular simulation allowed us to rationalize the activity profile of heteronemin against Hsp90, which provided valuable information for the further design of novel effective Hsp90 inhibitors.

## 3. Discussion

The structural features of the marine sesterpenoids, heteronemin, is interesting because it possesses a complex structure with several functional groups including a pentacyclic scalarane skeleton with a dihydrofuran moiety, nine stereogenic centers, a secondary hydroxy group at C-12, an acetal moiety at C-25, and a trisubstituted C-17–24 double bond. The unique arrangement of functionalities allowed several research groups to apply synthetic methodologies for its structural modification to improve its biological activity [10]. Our recent studies showed that heteronemin was effective in inhibiting tumor growth both in vitro and in vivo by inducing apoptosis and autophagy which suggested it could be further developed as a potential anticancer agent. When LNcap and PC3 cells were exposed to heteronemin at 2.56 μM for 24 h, the corresponding proliferation percentages were inhibited to 20.9 ± 0.6 and 52.7 ± 4.9%, respectively. Our results showed that cell growth of LNcap cells was significantly inhibited after heteronemin treatment in a dose-dependent manner with IC_50_ values of <2μM (Figure 1B). An in vivo antitumor activity of heteronemin was evaluated in animal xenograft model using human prostate cancer LNcap cells. The administration of heteronemin significantly diminished the growth of LNcap tumors compared with the solvent control after inoculation with cancer cells. Therefore, we focused on revealing the cytotoxic mechanism of action of heteronemin on LNcap cells throughout this study.

PhenylHydrazonoPyrazolone Sulfonate 1 (PHPS1) was identified as a specific inhibitor of protein tyrosine phosphatase Shp2 (PTPN11), which is a positive regulator of growth factor signal as the one of the key mechanisms in carcinogenesis and tumor progression [34,37,38]. PHPS1, a small molecule of PTP inhibitor, can significantly reduce cell proliferation and colony formation, indicating that the inhibition of Shp2 can suppress cancer cell proliferation in a Shp 2-dependent manner [34]. Our study showed that heteronemin treatment could activate PTP. In addition, the pretreatment with 50 μM of PHP promoted cell growth of LNcap cells about 28% compared with the control group and partially eliminated apoptosis and ROS generation induced by heteronemin treatment (2.56 μM) by 33.1% and 30.08%, respectively, indicating that PTP activation participated in signaling involved in the antitumor effect of heteronemin. Recent studies indicated that several inhibitors targeting protein tyrosine kinases provided enormous opportunities for drug development in cancer treatment [39,40,41]. Previous reports found that Shp2 is a positive regulator of cell growth, migration, and adhesion mediated with dephosphorylation of focal adhesion kinase (FAK), activation of Src kinase and the Ras/Erk1/2 pathway [41,42]. However, the role of Shp2 is still controversial because some PTPs are established as potential antitumor agents [43,44]. Here, we provided evidence for a novel mechanism linking PTP activation to the antitumor action of heteronemin through ROS generation, ER stress and inactivation of Rb2/TRADD pathway. The role of PTPs is intriguing and will be further studied for the development of potent anticancer therapy.

The expression of abnormal topoisomerase plays a critical role in chromosome instability and tumorigenesis which has been shown to be a proliferation marker associated with the tumor grade [45,46]. Currently, the topo II-targeting drugs can be divided into two categories including poisons such as etoposide, mitoxantrone, and doxorubicin and catalytic inhibitors such merbarone and aclarubicin [23]. Camptothecin (CPT) was clinically used for cancer treatment long before it was identified as a topo I inhibitor [47]. CPT produces severe side effects and has been replaced by more effective and safer topo I inhibitors including topotecan and irinotecan and there are other topo I inhibitors in the pharmaceutical pipeline [22,48]. Topoisomerase-targeting anticancer drugs act through topoisomerase poisoning which is associated with the development of secondary cancers and cardiotoxicity [49]. Thus, there is a need to develop more potent and efficient topo I and II inhibitors for cancer therapy. In the current study, we demonstrated that heteronemin is both topo I and II catalytic inhibitor with a cell-free system assay (Figure 4A,B). Our results suggested that heteronemin, the marine natural product, completely inhibited the catalytic reactions of topo I and IIα, as demonstrated by the cell-free system, and the topo IIα protein that was significantly depleted in heteronemin-treated LNcap cells in dose-dependent manner after 24 h (Figure 4C). A decrease in the related proteins’ expression is reflected in the suppression of the overall catalytic activity [24]. In consist with our previous study, heteronemin treatment completely inhibited the catalytic activity of topo I and IIα below the threshold levels, inhibiting DNA replication and, consequently, resulting in cell death [27]. In addition, the marine sesterterpenoid, heteronemin, could be as new potent candidate of topo II catalytic inhibitor for cancer therapy.

The molecular chaperone Hsp90 was a well-known modulator of conformation, stability and function of oncogenic proteins which play significant roles in the molecular mechanisms leading to cancer development and metastasis [50]. Accumulating evidence demonstrated that high expression of these Hsps is reported as a biomarker in array of cancers, such as breast, prostate, colorectal, lung, ovarian, gastric, oral, and esophageal cancer [51,52]. Thus, Hsps have been targeted for its clinically therapeutic potential. Hsp90 exists as a dimeric protein with each monomer containing an N-terminal ATP-binding domain, a middle cochaperone and client-binding domain, and a C-terminal dimerization domain [53]. Current evidence suggests that the mechanistic operation of several HSP90 inhibitors involves displacement of ATP, and thus, blockade of HSP90′s activity [54,55]. 17-allylamino-17-demethoxygeldanamycin (17-AAG) was the first Hsp90 inhibitor which entered in the clinical trial for BCR/ABL (Breakpoint Cluster Region/Abelson)-positive leukemia or HER2/NEU^+^ (Human Epidermal growth factor Receptor 2) breast cancer [56]. The scoring value of heteronemin was −8.97 kcal/mol, which was lower than −7.35 kcal/mol in 17-AAG, indicating that heteronemin had a more potent affinity to Hsp90 than 17-AAG with a molecular docking assay. Indeed, heteronemin attenuated intracellular expression of Hsp90 clients and inhibited the downstream signaling pathways, including IRAK1, *p*-Akt (Ser473) and XIAP, Rb2, HDAC1, PCNA and CDK4, phosphorylation of STAT3 (Ser727 and Tyr705). Consistence with previous studies [36,57,58], a host of pharmacodynamic biomarker, expression of Hsp70, increase in response to Hsp 90 inhibition (Figure 8B,C). Unfortunately, none of Hsp 90 inhibitor has been FDA-approved to date. The marine natural sesterterpenoids may be developed as the promising Hsp90 inhibitor for cancer therapy.

In the current study, we demonstrated that heteronemin, a marine sesterterpenoid derived the sponge *Hippospongia* sp., interferes with topo I and II as well as Hsp90 functions. Heteronemin exhibited potent cytotoxicity against both androgen-dependent and -independent type of human prostate cancer cells including PC3, LNcap and Du145 cancer cell lines [19]. Previous studies reported multiple biological activities of heteronemin, including antitubercular, cytotoxic, anti-intravasative activities [15,19,59,60]. Heteronemin induced renal cancer cells apoptosis and autophagy mediated by the suppression of Akt and MAPK pathway [14]. In addition, the marine natural product exhibited potent antitumor effect via the inhibition of c-Met/STAT3 pathway in prostate cancer PC3 cells [19]. Our current findings showed that the antitumor effect of heteronemin is manifested through apoptosis and autophagy by reducing viability, colony formation and tumor size. It also involved the induction of oxidative and ER stress. These results indicated that heteronemin might be used as a multitarget candidate for prostate cancer therapy.

## 4. Experimental Section

### 4.1. Bioassay Chemicals and Biological Materials

Cell lines were obtained from American Type Culture Collection (ATCC, Manassas, VA, USA). Cells were kept at 37 °C in 5% CO_2_ (humidified atmosphere). RPMI 1640 medium was used as the growing medium supplemented with 2 mM glutamine, 10% fetal calf serum and antibiotics (100 units/mL of penicillin and 100 μg/mL of streptomycin). Antibodies against AIF, PCNA, *p*-Stat 3 (Ser 727 and Tyr705), Rb 2, and TRADD were purchased from Cell Signaling Technologies (Beverly, MA, USA). Fetal calf serum (FCS), streptomycin, RPMI 1640 medium, trypan blue and penicillin G were obtained from GibcoBRL (Gaithersburg, MD, USA). Antibodies for topoisomerase I and II, CDK4, Chop, HDAC1, XIAP, acetyl-tubulin, *p*-Akt (Ser473), and *p*-HSF 1 were acquired from Santa Cruz Biotechnology (Santa Cruz, CA, USA). Anti-mouse and rabbit IgG peroxidase-conjugated secondary antibody were obtained from Pierce (Rockford, IL, USA). Annexin V-FITC/PI (propidium iodide) stain was purchased from Strong Biotech Corporation (Taipei, Taiwan). Dimethyl sulfoxide (DMSO), and 3-(4,5-dimethylthiazol-2-yl)-2,5-diphenyl-tetrazolium bromide (MTT) and all other chemicals were purchased from Sigma-Aldrich (St. Louis, MO, USA). Fluo-3, carboxy derivative of fluorescein (carboxy-H_2_DCFDA) and rhodamine 123 cationic dye were purchased from Molecular Probes and Invitrogen technologies (Carlsbad, CA, USA). Hybond ECL transfer membrane and ECL Western blotting detection kits were purchased from Amersham Life Sciences (Amersham, UK).

### 4.2. Stock Solution of Heteronemin

Heteronemin was separated from *Hippospongia* sp. and its chemical structure was determined by analyzing its spectroscopic data (1D and 2D NMR) and comparing those data to reported literature [11]. For the preparation of stock solution, heteronemin was dissolved in DMSO (Dimethyl sulfoxide) (20 μg/μL) and diluted before use.

### 4.3. MTT Cell Proliferation Assay

In the MTT [3-(4,5-dimethylthiazol-2-yl)-2,5-diphenyl-tetrazolium bromide] assay, culture plates (96-well) were used. After seeding the cells at at 4 × 10^4^ per well they were treated with several concentrations of the tested materials [61]. The cytotoxic effect of heteronemin was evaluated by MTT cell proliferation assay (thiazolyl blue tetrazolium bromide, Sigma-M2128) for 24, 48 or 72 h. To measure light absorbance values, ELISA reader (Anthoslabtec Instrument, Salzburg, Austria) was used at 570 and 620 nm (OD = OD_570_ − OD_620_). Calculations were performed to determine the concentration that caused 50% inhibition (IC_50_). MTT assay results were expressed as a percentage of the control ±SD obatined from *n* = 4 wells per experiment from three independent experiments.

### 4.4. Annexin V/PI Apoptotic Assay

Membrane integrity and phosphatidylserine (PS) externalization and were determined using annexin V-FITC (Fluorescein isothiocyanate) staining kit [61].After growing cells (10^6^) in 35-mm diameter plates, they were labeled with annexin V-FITC (10 μg/mL) and PI (20 μg/mL) before to harvesting. After labeling, all plates were washed with a binding buffer and then harvested. Cells at a concentration of 2 × 10^5^ cells/mL were resuspended using the binding buffer and then reassessed an FACS-Caliburflow cytometer (Beckman Coulter, Taipei, Taiwan) and the results were analyzed with CellQuest software (BD CellQuest Pro, Franklin Lakes, NJ, USA). For each measurement, approximately 10,000 cells were counted.

### 4.5. Determination of ROS Generation, Calcium Accumulation, and MMP Disruption 

Determination of MMP disruption and ROS generation were achieved as described in previous literature [61]. Calcium accumulation, MMP disruption, and ROS generation were evaluated with fluorescent calcium indicator (Fluo 3, 5 mM), rhodamine 123 cationic dye (5 μg/mL), and the carboxy derivative of fluorescein (carboxy-H_2_DCFDA, 1.0 mM), respectively. After treating cells with the tested compounds, they were labeled with a specific fluorescent dye for 30 min. After labeling, cells were washed with PBS and resuspended in PBS at a concentration of 1 × 10^6^ cells/mL. Cells were investigated using flow cytometry.

### 4.6. Immunofluorescence Analysis

Cells were treated with the tested compounds then fixed with 4% paraformaldehyde in 50 mM HEPES buffer (pH 7.3) for 30 min. 0.2% Trition X-100 in PBS (pH 7.4) was used to permeabilize cells for 20 min. Incubation of cells was done with 5% BSA in PBS containing 0.05% Trition X-100 (T-PBS) for 1 h. The experiment was done at room temperature to avoid non-specific protein binding. For 2 h, the cells were incubated with the primary Hsp70 antibodies (1:1000). This step was followed by secondary antibodies (Alexa Fluor 586-conjugated goat anti-mouse IgG (H + L) (Life Technologies, Carlsbad, CA, USA) diluted at 1:1000 for 1 h at room temperature. Before observation with FV1000 confocal laser scanning microscope (Olympus, Tokyo, Japan), cells were washed with PBS.

### 4.7. Topoisomerase I and II Catalytic Inhibitors and Poisons

The assays of topoisomerase I and II catalytic inhibitors and poisons were performed as described in literature [25,27]. Standard relaxation reaction mixtures (20 μL) containing 10 mM MgCl_2,_ 200 mM potassium glutamate, 50 mM Tris-HCl (pH 8.0), 50 μg/mL bovine serum albumin, 10 mM dithiothreitol, 0.3 μg of pHOT1 plasmid DNA, 1 mM ATP, two units of human topoisomerase II (Topogen, Columbus, OH, USA), the indicated concentrations of etoposide and heteronemin were incubated at 37 °C for 30 min. The termination of the reactions was done by adding 2 μL of 10% SDS to promote trapping the enzyme in a cleavage complex. This step was followed by the addition of 2.5 μL of proteinase K (50 μg/mL) to digest the bound protein, and the whole experiment was incubated at 37 °C for 15 min. Finally the reactions were stopped by adding 0.1 volume of the sample loading dye. Electrophoresis was used to analyze DNA products through vertical 2% agarose gels at 2 voltage/cm in 0.5× TAE buffer. Ethidium bromide was used to stain gels. The stained gels were photographed using an Eagle Eye II system (Stratagene, La Jolla, CA, USA). Quantitative analysis of DNA topo I and II activity was determined following reported literature [62].

### 4.8. Xenograft Animal Model with Human Prostate LNcap Cells

Xenograft was established in nude mice as described in previous literature [27]. The National Laboratory Animal and Research Center (Taipei, Taiwan) was the source of six-week-old male immunodeficient athymic mice. Mice were kept under standard laboratory conditions (temperature 24–26 °C, 12–12 h dark-light circle). Mice were fed with laboratory diet and water. Our study was approved by the Animal Care and Treatment Committee of Kaohsiung Medical University (IACUC Permit Number 101136). All experiments were conducted in strict adherence to the recommendations in the Guide for the Care and Use of Laboratory Animals of the National Institutes of Health. All efforts were made to minimize animal stress/distress. LNcap cells (1 × 10^6^) were suspended in 0.2 mL PBS then injected s.c. into the right flank of each mouse. Tumor growth was monitored every day. Mice with confirmed tumor growth after 14 days after tumor cell injection were randomly divided into two groups. The control group received only solvent, but the treated group received heteronemin (1.0 mg/kg) intraperitoneally. For every other day during 29 days, heteronemin was administrated. Carbon dioxide was used to kill the animals. For three times a week, the tumor size was measured using calipers. The tumor volumes were calculated according to the standard formula: width^2^ × length/2.

### 4.9. Molecular Docking

Autodock 4.2 with Lamarckian Genetic Algorithm were used for molecular docking [63]. From the protein data bank (http://www.rcsb.org/pdb/home/home.do), the target macromolecule, Hsp90 protein (PDB ID: 1YET), was obtained [64]. Small molecules, the cocrystallized protein substrates, including ligands, and water were removed. Polar hydrogens and Kallman united atom charges were added to the protein for docking calculation by AutoDock Tool 1.5.4 interfaces (ADT) [65]. MMFF94 force field in ChemBio3D software (version 11.0; Cambridge Soft Corp., St. Neots, UK) was applied to optimize ligands. To the ligand, polar hydrogens and Gasteiger charges were added to perform docking study by ADT. The AutoGrid program was used to calculate the grid box and was centered at the active site of Hsp90 with dimensions 56 × 56 × 56 Å grid points at a spacing of 0.375 Å. The size was big enough to allow the ligand to move freely in the search space. To the default setting, all docking parameters were set except for the following parameter: maximum number of energy evaluation increase to 25,000,000 per run. ADT was used to analyze the docking results. The results were shown by Accelrys Discovery Studio v3.5 client software (Accelrys Inc, San Diego, CA, USA (2005)).

### 4.10. Statistics

Our results were expressed as the mean ± standard deviation (SD). To compare each experiment, an unpaired Student’s *t*-test was used. A *p*-value of less than 0.05 was statistically significant.

## 5. Conclusions

The treatment of prostate cancer cells with the marine sesterterpenoid, heteronemin, induced mitochondrial dysfunction, oxidative and ER stresses resulting in apoptosis evidenced with annexin V/PI and JC-1 assay. Cell-free system and computational modeling using structure–function analysis supported that this compound can act as potential dual topoisomerase catalytic and Hsp90 inhibitors. The suppression of the expression of various Hsp90 client proteins in LNcap cells after treatment with heteronemin, was confirmed. The results also indicated that heteronemin is Hsp90 N-terminal inhibitor. In addition, our study provided insights on the antitumor mechanism of heteronemin and its clinical potential applications as a novel Hsp90 and topoisomerase II catalytic inhibitors to treat prostate patients. Sesterterpenoids represent interesting molecular architectures that bind preferentially to proteins involved in tumorigenesis. Recent advances in sesterterpenoids synthesis [36,66] open new avenues in their utilization in current therapeutic regimes.
